# Kawasaki disease shock syndrome: clinical characteristics and possible use of IL-6, IL-10 and IFN-γ as biomarkers for early recognition

**DOI:** 10.1186/s12969-018-0303-4

**Published:** 2019-01-05

**Authors:** Yandie Li, Qi Zheng, Lixia Zou, Jianqiang Wu, Li Guo, Liping Teng, Rongjun Zheng, Lawrence Kwok Leung Jung, Meiping Lu

**Affiliations:** 1 Department of Rheumatology Immunology and Allergy, Children’s Hospital, Zhejiang University School of Medicine, No.57, Zhugan Lane, Yan-an Road, Hangzhou, 310003 China; 2 Division of Rheumatology, Children’s National Medical Center, 111 Michigan Ave, NW, Washington, DC, 20010 USA

**Keywords:** Kawasaki disease, Shock, Cytokines, Clinical features

## Abstract

**Background:**

As an acute febrile and inflammatory disease, Kawasaki disease (KD) could develop Kawasaki disease shock syndrome (KDSS) sometimes. However its pathogenesis was still not well known. This study was to learn more about the clinical features and evaluate the role of cytokines in the pathogenesis of KDSS.

**Methods:**

We collected clinical and laboratory data retrospectively for all patients with KDSS(KDSS, *n* = 27)who were hospitalized at our hospital from Jan 2014 to Oct 2017. For patient with KDSS, we randomly identified 43 patients with KD as control subjects (KD, *n* = 43). Clinical features, laboratory evaluations were collected. Cytokines IL-2, IL-4, IL-6, IL-10, TNF-α and IFN-γ in serum were assayed using flow cytometric bead array.

**Results:**

The patients with KDSS were older age (43.41 ± 31.42 vs 28.81 ± 21.51 months, *P* < 0.05), longer duration of fever (10.63 ± 5.12 vs 6.98 ± 2.45 days, *P* < 0.05), higher WBC count, neutrophils, CRP, ESR, PCT and D-dimer, and lower hemoglobin and albumin, more severe hyponatremia and hypokalemia, more refractory to IVIG therapy, more coronary artery abnormalities (CAAs), aseptic meningitis, and longer duration of hospitalization than patients with KD (all *P* < 0.05). The levels of serum IL-6 [184.1 (27.7–2577.3) vs 54.1 (4–425) pg/ml], IL-10 [42.6 (5–236.7) vs 9.4 (3–94) pg/ml], TNF-α [2.6 (1.0–23.4) vs 2.1 (1–6) pg/ml] and IFN-γ [18.3 (4.5–94.4) vs 6.7 (2–56) pg/ml] in KDSS patients were significant higher than KD patients (all *P* < 0.05). ROC curves showed that 66.7 pg/ml of IL-6, 20.85 pg/ml of IL-10 and 8.35 pg/ml of IFN-γ had sensitivity and specificity for identifying KDSS as 85.2 and 62.8%; 66.7 and 83.7%; 74.1 and 74.4% respectively. No fatality was recorded in this series.

**Conclusions:**

KDSS were characteristic as more cytokine production and prone to developing IVIG non-responsiveness and CAAs. KD patients with IL-6 above 66.7 pg/ml, IL-10 above 20.85 pg/ml, and IFN-γ above 8.35 pg/ml suggested higher risk for KDSS.

## Background

Kawasaki disease (KD) is an acute, febrile vasculitis affecting children of younger than 5 years of age [1]. This disease often involved medium-sized arteries, especially coronary arteries [2]. 15–25% of untreated children will develop coronary artery abnormalities (CAAs) including coronary dilatation and aneurysms [3]. CAAs resulted from KD is an important cause of acquired heart disease in children [4]. Fatality rate decreased markedly after the introduction of IVIG therapy in the late 1980s. Timely initiation of treatment with IVIG has reduced the incidence of CAAs from 25 to 4% [5].

Sporadic cases of KD with a shock syndrome have been described in the literature as early as in the mid 1990s [6–8]. Dominguez recognized that a subgroup of children with KD were admitted to ICU with shock, often before recognized to have KD [9]. The concept of Kawasaki Disease Shock Syndrome (KDSS) was further defined by Kanegaye in 2009 [10]. They found that this syndrome was associated with more severe laboratory markers of inflammation and greater risk of CAAs, mitral regurgitation and prolonged myocardial dysfunction [11–15]. The incidence rate of KDSS varied from 2.60 to 6.95% in children in Western countries [9–11]. In contrast, studies in Taiwan reported lower incidence rate of 1.45% [12] and 1.9% [13]. However its etiology is still unknown.

Clinical characteristics of KDSS include poor perfusion or a shock-like state and typical signs of KD may not be obvious in the early phase of KDSS. So this syndrome is often difficult to diagnose [5]. Delayed treatment with IVIG increases the incidence of CAAs. KDSS patients need early aggressive management to reduce systemic and vascular inflammation. Therapeutic options include a repeat dose of IVIG, administration of infliximab (TNF-α blocker) [16], anakinra or methylprednisolone [17]. After standard treatment, some patients may still develop CAAs.

KD is a self-limiting disease, even in KD patients with severe CAAs or KDSS. During the self-limiting clinical course of KD, the intensity of systemic inflammation in the acute febrile stage of KD gradually increases and reaches a peak, after which the inflammation gradually decreases and the disease progresses to the convalescent stage. It has previously been postulated that the host immune reactions preceding this peak of inflammation process mediate tissue cell injury, whereas the immune reactions following the peak mediate tissue cell repair. The protein-homeostasis- system of the host is important for recovery from KD [18]. Pathogenesis of KDSS or other organ involvement in KD is unknown, but inflammatory mediators, including cytokines that are resulted from host immune reaction after an established infection of unknown pathogen(s), may be associated with KDSS and CAAs [18].

Cytokines may work mainly at cell level, and increased level of them may mean the numbers of immune cells involved in various pathologic lesions of KD. In a self-limiting disease like KD, pro-inflammatory reaction and counter-reaction of immune system act against insults from the disease and cytokines in plasma may be easily affected by stage of KD and severity of inflammation in KD. Therefore, study for cytokines’ role in KD is need in serial examinations during natural processes of the disease [19].

To document the distinguishing clinical features of this syndrome and the role of cytokines in its pathogenesis, we compared the clinical characteristics, treatment outcomes and levels of serum cytokines in children with KDSS or the children with KD but without the complicating shock syndrome.

## Materials and methods

### Patient and controls

From January 2014 to October 2017, 2203 children were diagnosed with KD in our hospital according to the standard diagnostic criteria [20, 21]. KDSS is a severe complication of KD was diagnosed by Kanegaye criteria [10]. Patients with the following findings were excluded from the study: (1) Sepsis with positive blood culture; (2) Other cardiovascular, hypotension or hypertension diseases, and primary disease associated with tumors, hematological diseases, congenital malformations, genetic metabolic diseases, primary myocarditis, primary diseases of major organs; (3) Relapsed KD patients requiring re-treatment; (4) incomplete collection of clinical and laboratory data. Echocardiography was done to determine whether CAAs were present [20, 21]. CAAs were defined using the criteria from the Japanese Ministry of Health [20]. Left ventricular (LV) dysfunction was defined by a shortening of the LV fraction < 28% and an associated lowering of the ejection fraction to < 54% [21]. IVIG-refractoriness was defined as persistent or recrudescence of fever < 48 h after completion of IVIG infusion (2 g/kg) [22, 23].

Of the 2203 patients with KD, 27 were identified as satisfying clinical criteria of KDSS [10]. For patient with KDSS, we randomly identified 43 patients with KD without shock syndrome as controlled subjects. We retrospectively evaluated the collected clinical and laboratory data from the patients with KDSS and the KD control subjects. This study was approved by the Research Ethics Committees of the Children’s Hospital Zhejiang University School of Medicine (2017-IRB-021) and is funded by Zhejiang basic public welfare research project (LGF19H100002).

### Clinical and laboratory data, echocardiography, and electrocardiogram collection

The following clinical data were collected: gender, age, race, clinical features and outcomes. Laboratory data were collected prior to treatment with IVIG. These included white blood cell count (WBC), C-reactive protein (CRP), erythrocyte sedimentation rate (ESR), procalcitonin (PCT), liver and kidney and coagulation function and other parameters. Echocardiography and electrocardiogram were collected as appropriate.

### Serum cytokine determination by flow cytometry

Serum cytokine determination was routinely done as part of a comprehensive laboratory evaluation in our hospital laboratory. All patients were tested for the six cytokines including IL-2, IL-4, IL-6, IL-10, TNF-α and IFN-γ at admission and at follow-up time points. Two milliliter(ml) of blood were collected, transferred to a serum separating tube and centrifuged at 1000 g at 20 °C for 20 min after clotting. The serum was carefully harvested, and was used to determine the cytokine level immediately, or stored at 2 °C to 8 °C until later for analysis (usually within 12 h). Concentrations of the six cytokines were quantitatively determined using the CBA Human Th1/Th2 Cytokine Kit II (BD Biosciences, San Jose, California) according to the manufacturer’s instructions. The minimum and maximum limits of detection for all six cytokines were 1 and 5000 pg/ml, respectively. The cytokine concentrations at the time of KD or KDSS diagnosis were used for analysis in this study.

The area under the ROC curve, referred to as the AUC, is an appropriate measure for describing the overall accuracy of a diagnostic test, with the higher AUC value signifying better diagnostic value. AUCs of IL-2, IL-4, IL-6, IL-10, TNF-α and IFN-γ were calculated respectively.

### Statistical analysis

Two-sample Student’s t-test was used to test for significant differences in the means of continuous variables. Mann-Whitney U test was used for comparing medians in skewed continuous variables. Proportions were compared using Fisher’s exact test appropriately. Receiver operating characteristic (ROC) curve analysis was used to select the optimal cutoff values of parameters. All statistical analyses were performed using SPSS version 20.0 (IBM, Armonk, NY, USA). A two-sided *P* value < 0.05 was considered statistically significant.

## Results

### Demographic and clinical features of patients with KDSS or KD

Twenty-seven of a total of 2203 patients with KD were identified as KDSS (1.23%). All patients were of Han descent in China. The patients with KDSS was older age (43.41 ± 31.42 vs 28.81 ± 21.51 months, *P* < 0.05), longer duration of fever(10.63 ± 5.12 vs 6.98 ± 2.45 days, *P* < 0.05) than those patients with KD. There was no significant difference in sex-distribution, clinical KD findings such as conjunctivitis, oral mucous membrane changes, cervical lymphadenopathy, changes in the peripheral extremity, polymorphous rash between 2 groups (all *P* > 0.05) (Table 1).

**Table 1:** Demographic and clinical features of patients with KDSS or KD

	KDSS(*n* = 27)	KD(*n* = 43)	*P value*
Age(months),			
Mean ± SD	43.41 ± 31.42	28.81 ± 21.51	0.04^a^
Median(range)	38 (2–102)	22 (3–89)	0.048^b^
Female gender, no. (%)	10(37.0)	24(55.8)	0.147^c^
Fever(days),mean ± SD	10.63 ± 5.12	6.98 ± 2.45	0.001 ^c^
Conjunctivitis, no.(%)	24(88.90)	41(95.3)	0.367 ^c^
oral mucous membrane changes, no.(%)	26(96.3)	41(95.3)	0.671 ^c^
Cervical lymphadenopathy,no.(%)	22 (81.5)	36(83.7)	0.526 ^c^
changes in the peripheral extremity, no.(%)	21 (77.8)	35(81.4)	0.764 ^c^
polymorphous rash, no.(%)	22 (81.5)	36(83.7)	0.526 ^c^

^a^Two-sample t test

^b^Manne-Whitney U test

^c^Fisher’s exact test

### Laboratory data of patients with KDSS or KD

Significantly higher WBC counts (19.04 ± 8.53 vs 13.12 ± 5.22 × 10^9^/L), PMNs (79.44 ± 17.56 vs 60.76 ± 14.34%), CRP (137.15 ± 54.80 vs 76.11 ± 50.66 mg/dl), ESR[101 (24–140) vs 67 (6–135) mm/h], PCT [2.33 (0.46–19) vs 0.56 (0.001–21.88) ng/ml] and lower hemoglobin (99.37 ± 12.69 vs 110.44 ± 10.17 g/L) were observed in patients with KDSS compared to patients with KD (all *P* < 0.05). Hypoalbuminemia (30.12 ± 6.54 vs 36.28 ± 3.73 mmol/L), hyponatremia (132.85 ± 3.73 vs 135.62 ± 3.68 mmol/L), hypokalemia (3.38 ± 0.50 vs 3.81 ± 0.46 mmol/L), higher level of ALT [53 (12–415) vs 18 (10–477) U/L], CK-MB (34.12 ± 16.05 vs 20.01 ± 8.46 mmol/L), creatinine [47.5 (34–456) vs 35 (25–58) μmol/L], BUN [5 (2–16) vs 2.62 (1.38–4.84) mmol/L], triglyceride (1.78 ± 0.60 vs 1.30 ± 0.51 mmol/L), and D-dimer [3.45 (1.13–7.88) vs 1.16 (0.36–1.43) mg/L] were found more frequently in patients with KDSS compared to patients with KD (all *P* < 0.05). Platelet count was lower but there was no statistical difference between groups (*P* > 0.05). Also, no statistical differences between groups were identified for lactic acid, amylase and fibrinogen (all *P* > 0.05)(Table 2). No known pathogens were identified in any of the study subjects.

**Table 2:** Comparison of laboratory data of patients with KDSS or KD

	KDSS (n = 27)	KD (n = 43)	*P value*
WBC count (×10^9^/L)	19.04 ± 8.53	13.12 ± 5.22	0.002^a^
PMNs (%)	79.44 ± 17.56	60.76 ± 14.34	< 0.001 ^a^
Hb(g/L)	99.37 ± 12.69	110.44 ± 10.17	< 0.001 ^a^
Platelet count (× 10^9^/L)	277(57–531)	355(150–629)	0.056 ^a^
ESR (mm/h)	101(24–140)	67(6–135)	0.041^b^
ESR > 40 mm/h,no(%)	25(92.59)	37(86.05)	0.472^c^
CRP (mg/dl)	137.15 ± 54.80	76.11 ± 50.66	< 0.001 ^a^
Albumin (mmol/L)	30.12 ± 6.54	36.28 ± 3.73	< 0.001 ^a^
Serum sodium concentration (mmol/L)	132.85 ± 3.73	135.62 ± 3.68	0.004 ^a^
Serum potassium concentration (mmol/L)	3.38 ± 0.50	3.81 ± 0.46	0.001 ^a^
ALT (U/L)	53 (12–415)	18 (10–477)	0.014^b^
AST (U/L)	43 (21–472)	35 (18–448)	0.080^b^
Lactic acid concentration (mmol/l)	1.96 ± 0.79	2.14 ± 0.81	0.372 ^a^
Creatinine(μmol/L)	47.5(34–456)	35(25–58)	< 0.001 ^b^
BUN(mmol/L)	5(2–16)	2.62(1.38–4.84)	< 0.001^b^
CK-MB(U/L)	34.12 ± 16.05	20.01 ± 8.46	< 0.001^a^
Triglyceride(mmol/L)	1.78 ± 0.60	1.30 ± 0.51	0.001 ^a^
Blood amylase (U/L)	54.3(11.9–404.8)	27.8 (7.1–75.5)	0.087^b^
Fibrinogen(g/L)	4(2–7)	4.46(3.87–5.12)	0.875^b^
D-dimer(mg/L)	3.45(1.13–7.88)	1.16(0.36–1.43)	0.001^b^
PCT(ng/ml)	2.33(0.46–19)	0.56(0.001–21.88)	< 0.001^b^

^a^Two-sample t test

^b^Manne-Whitney U test

^c^Fisher’s exact test

**Fig. 1:**
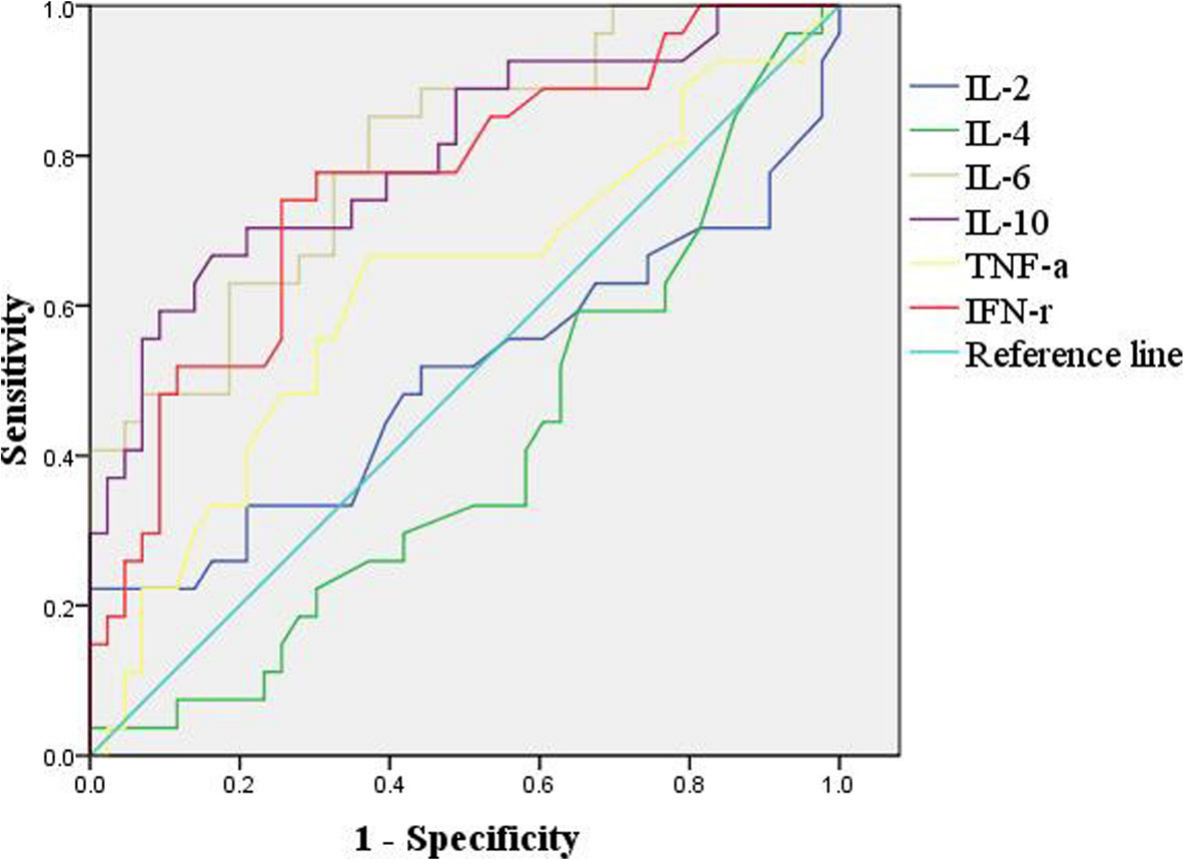
ROC curves of IL-2, IL-4, IL-6, IL-10, TNF-α, and IFN-γ between KD and KDSS. The diagonal line is the reference line

Significantly higher levels of serum IL-6 [184.1 (27.7–2577.3) vs 54.1 (4–425) pg/ml], IL-10 [42.6 (5–236.7) vs 9.4 (3–94) pg/ml], TNF-α [2.6 (1.0–23.4) vs 2.1 (1–6) pg/ml] and IFN-γ [18.3 (4.5–94.4) vs 6.7 (2–56) pg/ml] were found in patients with KDSS (all *P* < 0.05, respectively). There were no significant differences in IL-2 and IL-4 between two groups (all *P* > 0.05) (Table 3). The area under the ROC curves, referred to as the AUCs of IL-6, IL-10, IFN-γ, TNF-α, IL-2, and IL-4 levels were calculated to be 0.802, 0.801, 0.759, 0.618, 0.499, and 0.406, respectively, indicating that levels of IL-6, IL-10, and IFN-γ may be used as additional tools to distinguish KDSS from KD (P < 0.05). ROC curves showed that 66.7 pg/ml of IL-6,20.85 pg/ml of IL-10 and 8.35 pg/ml of IFN-γ had sensitivity and specificity for KDSS as 85.2 and 62.8%, 66.7 and 83.7%, 74.1 and 74.4% respectively. Thus, KD patients with IL-6 above 66.7 pg/ml, IL-10 above 20.85 pg/ml, and IFN-γ above 8.35 pg/ml may have a higher risk to evolve into KDSS (Fig. 1).

**Table 3:** Serum cytokine levels in patients with KDSS or KD

Cytokine(pg/ml)	KDSS (n = 27)	KD (n = 43)	*P value*
IL-2	4.2 (2.2–28.6)	4.1 (2–6)	0.947^b^
IL-4	3.2 (1.9–19.4)	3.9 (1.8–12)	0.168 ^b^
IL-6	184.1 (27.7–2577.3)	54.1 (4–425)	< 0.001^b^
IL-10	42.6 (5–236.7)	9.4 (3–94)	< 0.001^b^
TNF-ɑ	2.6 (1.0–23.4)	2.1 (1–6)	0.029^b^
IFN-γ	18.3 (4.5–94.4)	6.7 (2–56)	< 0.001^b^

^b^Manne-Whitney U test

### Treatment and outcomes of patients with KDSS or KD

All 27 KDSS patients (100%) received systemic steroids,fluid resuscitation and conventional therapy with aspirin and IVIG. Hypotension was recovered quickly after treatment in 14 patients. Other 13 (48.15%) of KDSS patients were admitted to the ICU due to hypotension or shock. Eight of cases (29.63%) required vasopressor including dobutamine, dopamine, and epinephrine because of persistent hypotension or shock. The other 5 patients can maintained normal blood pressure by intravenous normal saline. The hospital length of stay in patients with KDSS was significantly longer than KD patients (13.52 ± 5.12 vs 5.35 ± 2.02 days, *P* < 0.05). More common of IVIG-resistance (70.37% vs 2.30%), CAAs (55.56% vs 9.30%), and aseptic meningitis (48.15% vs 4.65%) were found in KDSS than KD (all *P* < 0.05). The male/female ratio for CAAs was 2:1 in KDSS. There were more other organ damage in KDSS group [85.19% (23/27) vs 14% (6/43), *P* < 0.05) including liver damage [15 (55.56%) vs 6 (14.00%), *P* < 0.05] and renal damage [8 (29.63%) vs 0 (0.00%), *P* < 0.05] The diagnosis of incomplete KD was more frequent in KDSS compared to KD group (29.62% vs 4.65%, *P* < 0.05). No fatality was seen in our study (Table 4).

**Table 4:** Comparison of treatment and outcome of patients with KDSS and KD

Outcome	KDSS (n = 27)	KD (*n* = 43)	*P value*
Hospitalized duration (days)			
mean ± SD	13.52 ± 5.12	5.35 ± 2.02	< 0.001^a^
median(range)	12(8-35)	5(3–15)	< 0.001^b^
CAAs, no(%)	15(55.56)	4(9.30)	< 0.001^c^
Aseptic meningitis, no. (%)	13(48.15)	2(4.65)	< 0.001 ^c^
Ejection fraction, %,(mean ± SD)	0.61 ± 0.07	0.64 ± 0.05	0.159 ^c^
ECG abnormality,no.(%)	18 (66.67)	2(4.65)	0.001 ^c^
IVIG-resistance,no.(%)	19 (70.37)	1(2.30)	0.001 ^c^
Second dose of IVIG,no(%)	10 (37.04)	2(4.65)	0.002 ^c^
ICU, no.(%)	13 (48.15)	0(0.00)	< 0.001 ^c^
vasopressor,no.(%)	8 (29.63)	0(0.00)	< 0.001 ^c^
Other organ damage,no. (%)	23 (85.19)	6(14.00)	0.001 ^c^
KD recurrence,no. (%)	2(7.41)	1 (2.3)	0.555^c^
Incomplete KD,no. (%)	8(29.62)	2(4.65)	0.010^c^
Death, no. (%)	0 (0.00)	0 (0.00)	1.000 ^c^

^a^Two-sample t test

^b^Manne-Whitney U test

^c^Fisher’s exact test

## Discussion

The strength of this study is the large number of KDSS patients that we can study. The incidence of KD in China has been shown to significantly higher than the Europeans [20, 24, 25]. In our study, the incidence rate of KDSS was 1.23%, which was lower than Western countries [9, 11], but in line with the Taiwanese studies and might suggest ethnic differences in the development of KDSS. The demographics of our patients with KDSS were similar to those of previous studies, with a trend towards older age [(43.41 ± 31.42) vs (28.81 ± 21.51) months] [10, 12–14]. The distribution of gender in KDSS differed in the various studies [11, 19]. In our study, the male/female ratio was 1.7:1 in KDSS group, and was similar to that reported [15].

Our study showed that it was difficult to distinguish KDSS from KD based only on clinical features (Table 1). Similar to previous studies [9–15, 26], the KDSS subjects tended to be older, higher incidence of CAAs and IVIG resistance, and had a longer duration of fever. As KDSS is characterized by shock, the need for vasopressor and ICU admission, more complications such as aseptic meningitis and other organ damage (Table 4). While these parameters do not help distinguish those KD patients who will develop KDSS and those who don’t, better biomarkers are needed.

During the acute phase of KD, the immune system is activated resulting in an increase in pro-inflammatory cytokines in the circulation. That these inflammatory cytokines may cause local and systemic damage has been supported by clinical findings and in the animal model [27–30]. In the Lactobacillus casei cell-wall extract (LCWE) induced mouse model of KD, IL-1 signaling pathways were involved in vasculitis, coronary arteritis and abdominal aortic aneurysm [31, 32]. Clinically, the inhibition of IL-1 was successful in treating refractory KD [33]. Together, these data demonstrated that elevated IL-1 level in KD may play a role in its pathogenesis [34]. Other inflammatory cytokines were found to be involved in pathogenesis of KD, both experimentally and clinically. Increased production of inflammatory cytokines (IL-1, TNF-α and IL-6) were associated with the pathogenesis of cardiac lesions in LCWE-induced KD murine model [35]. Similarly, increased inflammatory cytokine levels had been described in children with KD [29, 36, 37]. In our study, we found that the levels of cytokines including IL-6, IL-10, TNF-α and IFN-γ were significantly higher in sera of KDSS patients than the control KD patients. ROC curves showed that 66.7 pg/ml of IL-6, 20.85 pg/ml of IL-10 and 8.35 pg/ml of IFN-γ can differentiate KDSS from KD with sensitivity and specificity of 85.2 and 62.8%, 66.7 and 83.7%,74.1 and 74.4% respectively. This finding has not been previously reported in the literature. In fact, other cytokines also play an important role in the development of KDSS. We have not obtained similar results because of there are too few cases in our study.

Other inflammatory markers were also significantly elevated in KDSS patients. In our study, serum PCT levels were elevated in 9 KDSS patients who were initially diagnosed with septic shock. The symptom of shock was not relieved after antibiotic treatment, but improved after treatment with IVIG. Increased PCT have been reported to be associated with IVIG non-responsiveness [38, 39]. It has also been reported to be associated with CAAs in a study [40], although this finding was not corroborated in other studies [39, 41]. Thus, like the inflammatory cytokines, PCT was elevated in complications of KD including the shock syndrome. As PCT is produced by parenchymal cells of major organs [42], it may be a better biomarker to detect multi-organ failure, in contrast to CRP which is produced mainly in the liver [43]. Whether it can be used as a surrogate for the inflammatory cytokines remains to be determined.

Like the other studies, we did find that the platelet count in KDSS group tended to be lower (*P* = 0.056). CRP and the mean ESR in the KDSS group were higher. Our study differed from the other as we had found that the KDSS patients had higher number of WBC and PMNs, which was not described in other studies [9, 10, 13]. In our study, WBC, CRP, ESR and anemia were significantly higher in KDSS patients. These results have been shown to be closely related to elevated cytokine levels. For example, anemia was significantly worse in the KDSS patients which might be a result of IL-6 induced up-regulation of hepcidin [44]. Levels of CRP were positively correlated with IL-6 [45]. IL-6 promotes chronic inflammation which can lead to multiple organ damage and failure. With consistently elevated IL-6, hypoalbuminemia, hyponatremia and anemia appeared in patients [46]. Our study showed that the concentration of albumin, sodium and potassium in blood were significantly lower in KDSS group than KD patients and may reflect the significantly elevated cytokine such as IL-6 in these patients.

We believe that the increased in frequencies of CAAs and IVIG non-responsiveness in our KDSS patients were due to the increased inflammatory cytokines as well. Those patients who were IVIG non-responsive and those patients who developed CAAs had a higher serum level of IL-10 as compared to the patients who had not any complications [47, 48]. Significantly higher levels of IL-6 and IL-10 were seen in patients who were IVIG non-responders and in those who developed CAAs [29, 49]. Others have found that serum levels of sIL-2R and TNF-α were significantly higher in the patients with coronary aneurysm than in those without [50, 51]. Thus, mechanisms leading to the development of KDSS might be similar to the development of CAAs and IVIG non-responsiveness in KD. However, since not all subjects with IVIG non-responsiveness and CAAs developed shock syndrome, and since not all KDSS developed CAAs and IVIG non-responsiveness, additional factors may be involved in the pathophysiology of the shock syndrome.

One mechanism of action of IVIG may be due to the presence of naturally occurring anti-cytokine antibodies [52]. When there was excessive production of inflammatory cytokines as in the severely ill patients with KDSS, the amount of naturally occurring ant-cytokine antibodies contained in IVIG may be insufficient to block the excessive cytokines completely, so resulting in IVIG non-responsiveness in KDSS [52, 53]. Wang Y [29] suggested that KD patients with IVIG non-responsiveness had serum levels of IL-6 and IL-10 that decreased slowly and the levels of IL-4 and TNF-α that increased after treatment with IVIG. In our study, 70.37% of the KDSS patients were IVIG-nonresponders which was significant higher than 2.3% in KD group (Table 4).

It is clear that shock seen in KDSS is not due to infection. Non-infectious shock syndrome may be cardiac or non-cardiac in nature. We noted that CK-MB was significantly elevated in KDSS patients (Table 2). These data suggests that there may be local myocardial damage from myocardial and endocardial inflammation. This finding has also been reported by Inoue [54]. However, we found no difference in the ejection fraction between the 2 groups (Table 4), in contrast to those studies by Kanegaye and Taddio [10, 15]. The reason for this difference is not entirely clear.

Hypovolemia and shock may also result from vascular leak as exemplified by systemic capillary leak syndrome (SCLS) [55]. SCLS is reversible plasma extravasation and vascular collapse accompanied by hypoalbuminemia due to increased vascular leakage [56]. Interestingly this condition also responds to IVIG [57, 58]. In an in vitro system, Damle demonstrated that IL-2 activated lymphocytes could cause an increased flux of albumin across monolayer endothelial cells [59]. This suggested that IL-2 may play a role in the development of the SCLS [60]. The evidence that IL-2 production by T lymphocytes was downregulated by IVIG, provided a plausible explanation for the reversal of SCLS by IVIG [57]. The favorable response of KDSS to IVIG may be similar although we did not find evidence of increase IL-2 production in the KDSS patients (Table 3).

There are also shortcomings in this study. The number of cases in the control group is not enough, which may lead to sample bias. Since complete KD, incomplete KD, and KDSS are diagnosed by clinical manifestations, there could be a possibility of patient-selection bias. Although the control KD subsets could not match to KDSS group for age and fever duration, previous studies from the hematological department study group of our hospital showed that cytokine levels were more related to severe status of disease, but not to age and fever duration [61, 62].

## Conclusions

KDSS is an uncommon complication of KD but can lead to significant sequelae and poor outcome. The incidence of KDSS in our study was lower than the Western countries. KDSS were characteristic as hyperinflammation in serum and more prone to developing IVIG non-responsiveness and CAAs and aseptic meningitis. Increased production of IL-6, IL-10, TNF-α and IFN-γ cytokines may pay a key role in the pathogenesis of KDSS. IL-6 above 66.7 pg/ml, IL-10 above 20.85 pg/ml, and IFN-γ above 8.35 pg/ml suggested higher risk for KDSS. The paired cytokine studies may be helpful to define the cytokines’ role in KD and KDSS, but we did not analysis.

## Abbreviations

ALT: Alanine transaminase
AST: Aspartate aminotransferase
AUC: Area under ROC curve
BP: Blood pressure
BUN: Blood urine nitrogen
CAAs: Coronary artery abnormalities
CK-MB: Creatine kinase-MB
ESR: Erythrocyte sedimentation rate
IVIG: Intravenous Immunoglobulin
KD: Kawasaki disease
KDSS: Kawasaki Disease Shock Syndrome
PCT: Procalcitonin
PMN: Polymorphonuclear neutrophil
ROC: Receiver operating characteristic
SCLS: Systemic capillary leak syndrome
WBC: White blood cell

## References

[1] Nakamura Y, Yashiro M, Uehara R, Sadakane A, Chihara I, Aoyama Y (2010). Epidemiologic features of Kawasaki disease in Japan: results of the 2007–2008 Nationwide survey. J Epidemiol.

[2] Noto N, Kamiyama H, Karasawa K, Ayusawa M, Sumitomo N, Okada T (2014). Long-term prognostic impact of Dobutamine stress echocardiography in patients with Kawasaki disease and coronary artery lesions. J Am Coll Cardiol.

[3] Singh S, Sharma D, Bhattad S, Phillip S (2016). Recent advances in Kawasaki disease – proceedings of the 3rd Kawasaki disease summit, Chandigarh, 2014. Indian J Pediatr.

[4] Kobayashi T, Saji T, Otani T, Takeuchi K, Nakamura T, Arakawa H (2012). Efficacy of immunoglobulin plus prednisolone for prevention of coronary artery abnormalities in severe Kawasaki disease (RAISE study): a randomised, open-label, blinded-endpoints trial. Lancet.

[5] Takahashi K, Oharaseki T, Yokouchi Y, Yamada H, Shibuya K, Naoe S (2012). A half-century of autopsy results--incidence of pediatric vasculitis syndromes, especially Kawasaki disease. Circ J.

[6] Gamillscheg A, Zobel G, Karpf EF, Dacar D, Beitzke A, Stein JI (1993). Atypical presentation of Kawasaki disease in an infant. Pediatr Cardiol.

[7] Davies HD, Kirk V, Jadavji T, Kotzin BL (1996). Simultaneous presentation of Kawasaki disease and toxic shock syndrome in an adolescent male. Pediatr Infect Dis J.

[8] Senzaki H, Suda M, Noma S, Kawaguchi H, Sakakihara Y, Hishi T (1994). Acute heart failure and acute renal failure in Kawasaki disease. Acta Paediatr Jpn.

[9] Dominguez SR, Friedman K, Seewald R, Anderson MS, Willis L, Glode MP (2008). Kawasaki disease in a pediatric intensive care unit: a case-control study. Pediatrics.

[10] Kanegaye JT, Wilder MS, Molkara D, Frazer JR, Pancheri J, Tremoulet AH (2009). Recognition of a Kawasaki disease shock syndrome. Pediatrics.

[11] Gamez-Gonzalez LB, Murata C, Munoz-Ramirez M, Yamazaki-Nakashimada M (2013). Clinical manifestations associated with Kawasaki disease shock syndrome in Mexican children. Eur J Pediatr.

[12] Lin MT, Fu CM, Huang SK, Huang SC, Wu MH (2013). Population-based study of Kawasaki disease shock syndrome in Taiwan. Pediatr Infect Dis J.

[13] Chen PS, Chi H, Huang FY, Peng CC, Chen MR, Chiu NC (2015). Clinical manifestations of Kawasaki disease shock syndrome: a case-control study. J Microbiol Immunol Infect.

[14] Ma L, Zhang YY, Yu HG. Clinical manifestations of Kawasaki disease shock syndrome. Clin Pediatr (Phila). 2018;57(4):428–35. 10.1177/0009922817729483 28905639

[15] Taddio A, Rossi ED, Monasta L, Pastore S, Tommasini A, Lepore L (2017). Describing Kawasaki shock syndrome: results from a retrospective study and literature review. Clin Rheumatol.

[16] Singh S, Sharma D, Suri D, Gupta A, Rawat A, Rohit MK (2016). Infliximab is the new kid on the block in Kawasaki disease: a single-Centre study over 8 years from North India. Clin Exp Rheumatol.

[17] McCrindle BW, Rowley AH, Newburger JW, Burns JC, Bolger AF, Gewitz M (2017). Diagnosis, treatment, and long-term Management of Kawasaki Disease: a scientific statement for health professionals from the American Heart Association. Circulation.

[18] Lee KY, Rhim JW, Kang JH (2012). Kawasaki disease: laboratory findings and an immuno-pathogenesis on the premise of a “protein homeostasis system”. Yonsei Med J.

[19] Seo YM, Kang HM, Lee SC, Yu JW, Kil HR, Rhim JW (2018). Clinical implications in laboratory parameter values in acute Kawasaki disease for early diagnosis and proper treatment. Korean J Pediatr.

[20] Newburger JW, Takahashi M, Gerber MA, Gewitz MH, Tani LY, Burns JC (2004). Diagnosis, treatment, and long-term management of Kawasaki disease: a statement for health professionals from the committee on rheumatic fever, endocarditis and Kawasaki disease, council on cardiovascular disease in the young, American Heart Association. Circulation.

[21] Adler AC, Kodavatiganti R (2016). Kawasaki disease with Giant coronary aneurysms requiring a ventricular assist device to separate from extracorporeal membrane oxygenation: coronary issues can be a pediatric problem too!. A A Case Rep.

[22] Saneeymehri S, Baker K, So TY (2015). Overview of pharmacological treatment options for pediatric patients with refractory Kawasaki disease. J Pediatr Pharmacol Ther.

[23] Ayusawa M (2014). Clinical course and features in acute stage of Kawasaki disease. Nihon Rinsho.

[24] Uehara R, Belay ED (2012). Epidemiology of Kawasaki disease in Asia, Europe, and the United States. J Epidemiol.

[25] Chen JJ, Ma XJ, Liu F, Yan WL, Huang MR, Huang M (2016). Epidemiologic features of Kawasaki disease in Shanghai from 2008 through 2012. Pediatr Infect Dis J.

[26] Gatterre P, Oualha M, Dupic L, Iserin F, Bodemer C, Lesage F (2012). Kawasaki disease: an unexpected etiology of shock and multiple organ dysfunction syndrome. Intensive Care Med.

[27] Lin YJ, Chang JS, Liu X, Lin TH, Huang SM, Liao CC (2013). Sorting nexin 24 genetic variation associates with coronary artery aneurysm severity in Kawasaki disease patients. Cell Biosci.

[28] Si F, Wu Y, Gao F, Feng S, Liu R, Yi Q (2017). Relationship between IL-27 and coronary arterial lesions in children with Kawasaki disease. Clin Exp Med.

[29] Wang Y, Wang W, Gong F, Fu S, Zhang Q, Hu J (2013). Evaluation of intravenous immunoglobulin resistance and coronary artery lesions in relation to Th1/Th2 cytokine profiles in patients with Kawasaki disease. Arthritis Rheum.

[30] Gitiaux C, Kossorotoff M, Bergounioux J, Adjadj E, Lesage F, Boddaert N (2012). Cerebral vasculitis in severe Kawasaki disease: early detection by magnetic resonance imaging and good outcome after intensive treatment. Dev Med Child Neurol.

[31] Lee Y, Wakita D, Dagvadorj J, Shimada K, Chen S, Huang G (2015). IL-1 signaling is critically required in stromal cells in Kawasaki disease Vasculitis mouse model: role of both IL-1alpha and IL-1beta. Arterioscler Thromb Vasc Biol.

[32] Wakita D, Kurashima Y, Crother TR, Noval RM, Lee Y, Chen S (2016). Role of Interleukin-1 signaling in a mouse model of Kawasaki disease-associated abdominal aortic aneurysm. Arterioscler Thromb Vasc Biol.

[33] Dusser P, Kone-Paut I (2017). IL-1 inhibition may have an important role in treating refractory Kawasaki disease. Front Pharmacol.

[34] Cohen S, Tacke CE, Straver B, Meijer N, Kuipers IM, Kuijpers TW (2012). A child with severe relapsing Kawasaki disease rescued by IL-1 receptor blockade and extracorporeal membrane oxygenation. Ann Rheum Dis.

[35] Okitsu-Negishi S, Nakano I, Suzuki K, Hashira S, Abe T, Yoshino K (1996). The induction of cardioangitis by lactobacillus casei cell wall in mice. I. the cytokine production from murine macrophages by lactobacillus casei cell wall extract. Clin Immunol Immunopathol.

[36] Lin CY, Lin CC, Hwang B, Chiang BN (1991). The changes of interleukin-2, tumour necrotic factor and gamma-interferon production among patients with Kawasaki disease. Eur J Pediatr.

[37] Lee SB, Kim YH, Hyun MC, Kim YH, Kim HS, Lee YH (2015). T-helper cytokine profiles in patients with Kawasaki disease. Korean Circ J.

[38] Gowin E, Wysocki J (2016). Limited diagnostic value of procalcitonin in early diagnosis of adult onset Still's disease. Reumatologia.

[39] Yoshikawa H, Nomura Y, Masuda K, Koriya C, Arata M, Hazeki D (2012). Serum procalcitonin value is useful for predicting severity of Kawasaki disease. Pediatr Infect Dis J.

[40] Okada Y, Minakami H, Tomomasa T, Kato M, Inoue Y, Kozawa K (2004). Serum procalcitonin concentration in patients with Kawasaki disease. J Inf Secur.

[41] Catalano-Pons C, Andre MC, Chalumeau M, Guerin S, Gendrel D (2007). Lack of value of procalcitonin for prediction of coronary aneurysms in Kawasaki disease. Pediatr Infect Dis J.

[42] Pfafflin A, Schleicher E (2009). Inflammation markers in point-of-care testing (POCT). Anal Bioanal Chem.

[43] Muller B, White JC, Nylen ES, Snider RH, Becker KL, Habener JF (2001). Ubiquitous expression of the calcitonin-i gene in multiple tissues in response to sepsis. J Clin Endocrinol Metab.

[44] Kuo HC, Yang YL, Chuang JH, Tiao MM, Yu HR, Huang LT (2012). Inflammation-induced hepcidin is associated with the development of anemia and coronary artery lesions in Kawasaki disease. J Clin Immunol.

[45] Mitani Y, Sawada H, Hayakawa H, Aoki K, Ohashi H, Matsumura M (2005). Elevated levels of high-sensitivity C-reactive protein and serum amyloid-a late after Kawasaki disease: association between inflammation and late coronary sequelae in Kawasaki disease. Circulation.

[46] Schmidt-Arras D, Rose-John S (2016). IL. 6 pathway in the liver: from physiopathology to therapy. J Hepatol.

[47] Weng KP, Hsieh KS, Hwang YT, Huang SH, Lai TJ, Yuh YS (2010). IL-10 polymorphisms are associated with coronary artery lesions in acute stage of Kawasaki disease. Circ J.

[48] Guo MM, Tseng WN, Ko CH, Pan HM, Hsieh KS, Kuo HC (2015). Th17- and Treg-related cytokine and mRNA expression are associated with acute and resolving Kawasaki disease. Allergy.

[49] Xie T, Wang Y, Fu S, Wang W, Xie C, Zhang Y (2017). Predictors for intravenous immunoglobulin resistance and coronary artery lesions in Kawasaki disease. Pediatr Rheumatol Online J.

[50] Teraura H, Kotani K, Minami T, Takeshima T, Shimooki O, Kajii E (2017). The serum concentration of soluble interleukin-2 receptor in patients with Kawasaki disease. Ann Clin Biochem.

[51] Hu P, Jiang GM, Wu Y, Huang BY, Liu SY, Zhang DD (2017). TNF-alpha is superior to conventional inflammatory mediators in forecasting IVIG nonresponse and coronary arteritis in Chinese children with Kawasaki disease. Clin Chim Acta.

[52] Watanabe M, Uchida K, Nakagaki K, Trapnell BC, Nakata K (2010). High avidity cytokine autoantibodies in health and disease: pathogenesis and mechanisms. Cytokine Growth Factor Rev.

[53] Ikeda K, Mizoro Y, Ameku T, Nomiya Y, Mae SI, Matsui S (2016). Transcriptional analysis of intravenous immunoglobulin resistance in Kawasaki disease using an induced pluripotent stem cell disease model. Circ J.

[54] Inoue Y, Kobayashi T, Tomomasa T, Morikawa A (1999). Macro creatine kinase in Kawasaki disease. Pediatr Cardiol.

[55] Pineton DCM, Luyt CE, Beloncle F, Gousseff M, Mauhin W, Argaud L (2017). The clinical picture of severe systemic capillary-leak syndrome episodes requiring ICU admission. Crit Care Med.

[56] Park S, Eun LY, Kim JH (2017). Relationship between serum sodium level and coronary artery abnormality in Kawasaki disease. Korean J Pediatr.

[57] Lambert M, Launay D, Hachulla E, Morell-Dubois S, Soland V, Queyrel V (2008). High-dose intravenous immunoglobulins dramatically reverse systemic capillary leak syndrome. Crit Care Med.

[58] Shin JI, Lee JS (2009). Beneficial effect of intravenous immunoglobulins on systemic capillary leak syndrome in patients with monoclonal gammopathy. Crit Care Med.

[59] Damle NK, Doyle LV, Bradley EC (1986). Interleukin 2-activated human killer cells are derived from phenotypically heterogeneous precursors. J Immunol.

[60] Modiano JF, Amran D, Lack G, Bradley K, Ball C, Domenico J (1997). Posttranscriptional regulation of T-cell IL-2 production by human pooled immunoglobin. Clin Immunol Immunopathol.

[61] Chen YY, Wang ZJ, Luo ZB, Zhao N, Yang SL, Tang YM (2016). Comparison of Th1/Th2 cytokine profiles between primary and secondary haemophagocytic lymphohistiocytosis. Ital J Pediatr.

[62] Luo ZB, Chen YY, Xu XJ, Zhao N, Tang YM (2017). Prognostic factors of early death in children with hemophagocytic lymphohistiocytosis. Cytokine.

